# Influence of Enzyme Supplementation in the Diets of Broiler Chickens Formulated with Different Corn Hybrids Dried at Various Temperatures

**DOI:** 10.3390/ani11030643

**Published:** 2021-02-28

**Authors:** Franciele C. N. Giacobbo, Cinthia Eyng, Ricardo V. Nunes, Cleison de Souza, Levy V. Teixeira, Rachel Pilla, Jan S. Suchodolski, Cristiano Bortoluzzi

**Affiliations:** 1 Ciências Agrárias, Universidade Estadual do Oeste do Paraná, 85960-000 Mal. C. Rondon, Brazil; frannavarini@hotmail.com (F.C.N.G.); nunesrv@hotmail.com (R.V.N.); cleisondsz@hotmail.com (C.d.S.); 2 DSM Nutritional Products, 04551-065 São Paulo, Brazil; levy.teixeira@dsm.com; 3 Department of Small Animal Clinical Sciences, Texas A&M University, College Station, TX 77843, USA; rpilla@cvm.tamu.edu (R.P.); jsuchodolski@cvm.tamu.edu (J.S.S.); 4 Department of Poultry Science, Texas A&M University, College Station, TX 77845, USA; bortoluzzi.c@gmail.com

**Keywords:** amylase, cecal microbiota, drying temperature, protease, xylanase

## Abstract

**Simple Summary:**

The use of exogenous enzymes is a common nutrition strategy of the poultry industry. However, the influence of this additive on the microbiota and its efficiency when the diets are formulated with different hybrids of corn dried under high temperature are still unclear. From a practical point of view, evaluating the mode of action of enzymes in different situations is crucial to ensure competitive performance results with low production costs. The current study confirmed that regardless of corn hybrids and drying temperature, dietary supplementation with amylase, xylanase, and protease was beneficial for intestinal morphology and allowed a modulation of the cecal microbiota. This influence may have changed the digestive process and use of nutrients by the broilers, resulting in better animal performance.

**Abstract:**

We evaluated the influence of enzymatic supplementation on the growth performance and cecal microbiota of broilers. A total of 2160 1-day-old male chicks were used in a 3 × 2 × 2 factorial arrangement (three corn hybrids, two drying temperatures −80 and 110 °C, with or without the inclusion of an enzymatic blend (amylase, xylanase, and protease) (20 birds/pen, *n* = 9). For all performance and digestibility parameters, we observed, in general, isolated effects of the corn hybrids and drying temperature. Birds that received the enzymatic blend in the diet showed better weight gain from 1 to 21 days (d) and better digestibility coefficients of nutrients at 42 d. Birds fed diets with corn dried at 80 °C showed a better feed conversion ratio from 1 to 42 d. At 21 d of age, enzymatic supplementation had positive effects on jejunum morphology. Enzyme supplementation increased the abundance of the phylum Tenericutes, class Bacilli and Mollicutes, reduced Clostridia, and increased the abundances of the families Lactobacillaceae, Anaeroplasmataceae, and O_RF39;F. In conclusion, the addition of amylase, xylanase, and protease led to a better nutrient digestibility, performance, and intestinal morphology. In addition, enzyme supplementation changed the diversity, composition, and predicted function of the cecal microbiota at d 21.

## 1. Introduction

Corn is one of the main ingredients in poultry diets due to its high energy content. According to the Brazilian Tables of Poultry and Swine [1], corn contains an average of 73.5% starch, 9% protein, 4.3% lipids and 1.5% minerals. However, climatic conditions, genetics, processing, and post-harvest storage may alter the chemical composition of the grain and, consequently, its nutritional quality, mainly due to changes in the starch structure, lipids, and proteins [2,3]. Among the post-harvest processes, the temperature used to reduce the water content of the corn grain for further storage is often determined by the production flow and may reach levels above 100 °C. The adverse effects related to the use of high temperatures during the drying process include the denaturation of vitamins and proteins as well as a reduction in the digestibility of the protein due to the Maillard reaction and the formation of retrograded starch [4]. Kaczmarek et al. [5] and Bhuiyan et al. [6] observed changes in the digestibility of starch and protein when corn was dried at temperatures above 100 °C. This reduction in digestibility may be related to the nature and solubility of proteins, which may affect the use of nutrients, lower the availability of amino acids, and impair the action of endogenous α-amylase [7]. Kaczmarek et al. [5] obtained lower weight gain and worse feed conversion ratio in broilers fed diets formulated with corn dried under high temperature (140 °C).

Among the available strategies to improve nutrient use, the dietary inclusion of exogenous enzymes has been the focus in poultry nutrition. Depending on the ingredient, the amount of antinutritional factors varies, which may impair the adequate nutrient use. The cellular wall contains significant quantities of non-starch polysaccharides (NSP) that cannot be hydrolyzed by the normal digestive processes of broilers due to the lack of specific enzymes [8]. It is estimated that 400 to 450 kcal of energy per kg of feed remain undigested in the digestive tract of broilers due to the NSP present in the diets formulated based on corn and soybean meal [9]. The degradation of the cell wall may provide more energy to the animal and increase access to other enzymes such as proteases to the cellular content, which may increase the overall digestibility [10]. In addition, a mechanism of action related to the changes of the intestinal microbiota due to the use of enzymes must be emphasized [5]. The use of exogenous enzymes favors the breakdown of dietary components that may be used for the proliferation of beneficial bacteria [11], which may also have an effect on nutrient digestibility and immune modulation [12].

On the other hand, the potential of exogenous enzymes in releasing higher amounts of nutrients in diets formulated with different hybrids of corn dried under high temperature is still unclear. Additionally, studies that relate the mechanism of action of enzymes on the microbiota and its influence on the digestive processes are scarce. In this context, we hypothesized that the drying process and different hybrids of corn may change the chemical composition and the use of nutrients present in the corn grain and that the inclusion of dietary enzymes minimizes the deleterious effects of artificial drying. The objective of the present study was to determine the effects of different hybrids of corn and drying temperature as well as the inclusion of dietary enzymes on the growth performance, carcass yield, intestinal morphology, and ileal digestibility of broiler chickens. We also evaluated the effect of enzymatic supplementation on the cecal microbiota.

## 2. Materials and Methods

### 2.1. Housing, Birds and Treatments

The experiment was conducted at the Poultry Research Center at the Western State Paraná University, Marechal Cândido Rondon, Paraná state, Brazil. All the procedures were approved by the Institutional Animal Care and Use Committee (protocol #06/19).

A total of 2160 one-day-old male Cobb 500^®^ broiler chickens were used in the experiment. Chickens were weighed and allocated in a completely randomized design, with 12 experimental treatments, and nine replicates of 20 birds each. The birds had ad libitum access to water and feed formulated according to Rostagno et al. [13]. The diets were isocaloric and isoproteic and divided into starter (1 to 7 d), grower (8 to 21 d), and finisher (22 to 42 d), and provided in mash form (Table 1). The lighting program followed the strain recommendations. The temperature control of the poultry barn was achieved using electric heaters, exhaust fans, and pad cooling. The maximum and minimum registered temperatures were 33.4 and 20.7 °C, respectively. The birds were placed on a new litter of wood shavings.

The experimental treatments were arranged in a 3 × 2 × 2 factorial scheme with three hybrids of corn, two drying temperatures (80 and 110 °C), and with or without the inclusion (on top) of an enzymatic blend. The enzymatic blend was added to diets according to the manufacturer’s instructions and contained 80 KNU/kg of amylase (RONOZYME^®^ HiStarch; DSM Nutritional Products, São Paulo, Brazil), 100 FXU/kg of xylanase (RONOZYME^®^ WX; DSM Nutritional Products, São Paulo, Brazil), and 15,000 PROT/kg of protease (RONOZYME^®^ ProAct; DSM Nutritional Products, São Paulo, Brazil). All diets were formulated with the inclusion of phytase (1000 FYT/kg of feed, RONOZYME^®^ HiPhos; DSM Nutritional Products, São Paulo, Brazil), considering the matrix value of 0.15% for total calcium and available phosphorus.

### 2.2. Enzymatic Blend

RONOZYME^®^ HiStarch is produced from the submerged fermentation of *Bacillus licheniformis* containing 600 KNU/kg. One KNU (kilo-Novo α-amylase units) is the amount of enzyme that releases, from a two-step reaction, 6 mol of nitrophenol per minute from 1.86 mM of ethylidene-G7-p-nitrophenyl-maltoheptaose at pH 7.0 and 37 °C. RONOZYME^®^ WX is an endo–1,4–beta–xylanase produced from a genetically modified strain of *Aspergillus oryzae.* The enzyme activity of xylanase in fungal β- xylanase units (FXU) is defined as the amount of enzyme that releases 7.8 µmol of reducing sugars (xylose equivalents) of azo wheat arabinoxylan per minute at pH 6.0 and 50 °C. RONOZYME^®^ ProAct is manufactured from the fermentation of *Bacillus licheniformis* containing *Nocardiopsis prasina* with a transcribed gene, being considered a monocomponent protease with 75,000 unit of protease/kg. The activity of this enzyme (PROT) is defined as the amount of enzyme needed to degrade 1 µmol of p-nitroanilide of substrate 1 µmol (Suc-Ala-Ala-Pro-Fen-N-succinyl Ala-Ala-Pro-Fen-p-nitroanilide) per minute at pH 9.0 and 37 °C. Ultimately, RONOZYME^®^ Hiphos is a 6-microbial phytase expressed by synthetic genes of *Aspergillus oryzae* that has an activity of 10,000 FYT/kg. One unit of phytase (FYT) is the amount of enzyme necessary to release 1 µmol of inorganic phosphate under regular conditions (acetate buffer 0.25 M, pH 5.5, temperature of 37 °C and five μmol of sodium phytate).

### 2.3. Hybrids of Corn

The three hybrids of corn evaluated herein were classified as semi-hard grain for hybrid 1 (30A37 PW—Morgan) and 2 (30A77 PW—Morgan) and semi-dentate for hybrid 3 (DKB 330 PRO—Dekalb). All the corn hybrids were produced under similar conditions, in a region located in Western Paraná State, Brazil, with the objective to reduce any interference of soil and climate on the chemical characteristics of the grain.

The corn grains were harvested with 23% of moisture and dried under 80 or 110 °C using a mixed-flow grain dryer. The drying process was concluded when the moisture reached 14%, with the water content of the grain being determined throughout the duration of the process using an electronic meter, model 919FOB (MOTOMCO, Porto Alegre, RS, Brazil). The average time of the drying process was four hours when the grains were dried at 110 °C and six hours when dried at 80 °C.

According to the analysis conducted by proximal infrared reflectance spectrophotometry (NIRS) using FT-NIR-TANGO equipment (Bruker, MA, USA), the average levels, independently of the dry temperature, of dry matter (DM), crude protein (CP), ether extract (EE), mineral matter (MM), and gross energy (GE) were 87.45, 7.65, 4.07, 1.12%, and 4135 kcal/kg for hybrid 1; 88.40, 7.93, 4.07, 1.02%, and 4132 kcal/kg for hybrid 2; and 87.25, 8.10, 3.96, 1.10%, and 4056 kcal/kg for hybrid 3, respectively. According to the methodology of Silva and Queiroz [14] for neutral detergent fiber and the method of Englyst et al. [15] for total NSP of the corn samples, independently of the drying temperature, respectively, values of 11.45% and 7.13% for hybrid 1; 11.85% and 6.55% for hybrid 2; and 10.75% and 6.35% for hybrid 3 were observed.

### 2.4. Sample Collection and Analyses Performed

The birds and feed were weighed on d 7, 21, and 42 to determine body weight gain (BWG), feed intake (FI), and feed conversion ratio (FCR) for each treatment. Mortality was checked daily to correct FI and FCR according to Sakomura and Rostagno [16]. On d 21 and 42, one bird within the average weight (±5%) per experimental unit was selected and euthanized by electronarcosis followed by bleeding to collect jejunum for morphology analysis. Additionally, on d 21, the cecal content of three birds per pen was collected, squeezed into a 50 mL tube, and immediately frozen at −20 °C for further microbiota analysis. Ileal digestibility and carcass yield were evaluated on d 42. 

### 2.5. Intestinal Morphology

The jejunum morphology was determined from a 2 cm segment collected between the distal duodenal loop and the Meckel diverticulum. The segment was collected, longitudinally opened, rinsed with a saline solution, and fixed in a 10% neutral buffer formalin solution. Subsequently, the tissue was dehydrated and immersed into paraffin, according to Luna [17]. Following the semi-serial microtomy, at a thickness of 5 µm, the histological sections were stained using the hematoxylin-eosin technique.

The morphological analyses were performed using the image system (Pro plus Image 4.1, Media Cybernetics, Silver Spring, MD, USA). For each slide, we measured the height of 30 villus (from the tip of the villus to the villus-crypt junction), 30 crypts depth (from villus-crypt junction to the base of the crypt), and the width of 30 villi and 30 crypts (the distance from the outside epithelial edge to the outside of the opposite epithelial edge along a line passing through the vertical midpoint of the villus or crypt) [18]. These measures were taken to calculate the area of absorption (AA) surface of the intestinal mucosa, through the equation proposed by Kisielinski et al. [19]. In addition, the villus:crypt ratio (V:C) was calculated by dividing villus height (VH) by crypt depth (CD).

### 2.6. Cecal Microbiota

In order to evaluate the cecal microbiota, the initial treatments were divided into two groups: with or without the inclusion of the enzymatic blend, which totaled nine samples per treatment composed of a poll of cecal content of six birds each. The cecal content was sent to the Neoprospecta Microbiome Technologies where the bacterial DNA was isolated and submitted to high-throughput sequencing of the 16S rRNA V3/V4 region with a proprietary protocol (Neoprospecta Microbiome Technologies, Canasvieiras, SC, Brazil). The amplification of the 16S rRNA V3/V4 region was carried out using the 341F (CCTACGGGRSGCAGCAG) [20] and 806R (GGACTACHVGGGTWTCTAAT) [21] primers. The 16S rRNA libraries were sequenced using the MiSeq Sequencing System (Illumina Inc., San Diego, CA, USA) with the V2 kit, 300 cycles, single-end sequencing.

The sequences were processed and analyzed using a Quantitative Insights Into Microbial Ecology 2 (QIIME 2) [22] v 2019.7 pipeline. The raw sequences were uploaded to the National Center for Biotechnology Information (NCBI) Sequence Read Archive under the project number PRJNA613080. Briefly, the sequences were demultiplexed and the Amplicon Sequence Variant (ASV) table was created using DADA2 [23]. Prior to downstream analysis, sequences assigned as chloroplast, mitochondria, and low abundance ASVs, containing less than 0.01% of the total reads in the dataset, were removed. All samples were rarefied to even sequencing depth, based on the lowest read depth of samples, to 20,568 sequences per sample.

To estimate the metabolic pathways affected by enzyme supplementation, Phylogenetic Investigation of Communities by Reconstruction of Unobserved States (PICRUSt) [24] was calculated using the default PICRUSt2 pipeline. Alpha diversity was measured with the Chao1 (richness), and Shannon diversity indices. Beta diversity was evaluated with the unweighted phylogeny based UniFrac [25] distance metric and visualized using a principal coordinate analysis (PCoA) plot.

### 2.7. Ileal Digestibility

The digestibility of nutrients was determined at the end of the experimental period (42 d). The insoluble ash source (Celite™; Lompoc, CA, USA) was added to the experimental diets at the rate of 1% as an undigestible marker, wherein the birds were submitted to seven days of adaptation to the diet before collecting the samples. At 42 d of age, two birds per experimental unit were euthanized by electronarcosis, and the ileal digesta from the posterior half between Meckel’s diverticulum and 2 cm prior to the ileo-ceco-colonic-junction was collected. The digesta was then homogenized, weighed, and vacuum freeze dried at −40 °C for 72 h (Liotop, L101, Liobrás, São Carlos, Brazil).

After drying, the samples were ground and sent to the Animal Nutrition Laboratory of the university. Dry matter, gross energy (GE), nitrogen (N), mineral matter, and acid-insoluble ash (AIA) were determined. Nitrogen was analyzed by the Kjeldahl method, and the DM was in accordance with Silva and Queiroz [14]. For the determination of GE, the samples were pelleted and analyzed in a calorimetric bomb (model C200, IKA Works Inc., Wilmington, NC, USA). Acid-insoluble ash, present in the feed and digesta samples, was determined by the methodology proposed by Van Keulen and Young [26]. The digestibility coefficient of the DM, CP, MM, and digestible energy were determined according to Sakomura and Rostagno [16].

### 2.8. Carcass Yield

At the end of the experimental period (42 d), 18 birds per treatment, within the mean weight (±5%), were selected, individually weighed, and euthanized by electronarcosis followed by bleeding to determine carcass and cuts yields. For carcass yield, the weight of the eviscerated carcass (without head, feet, neck, and abdominal fat) was considered in relation to the live weight before slaughter, and the for cuts yield (breast, wings, and legs), the eviscerated carcass weight was taken into consideration. The percentage of abdominal fat was determined using the weight of the fat present around the cloaca, gizzard, proventriculus, and adjacent abdominal muscles in relation to the weight of the live bird.

### 2.9. Statistical Analysis

The normality of the data was checked using the Shapiro–Wilk test. The data were then analyzed as a 2-way ANOVA using the GLM procedure of the SAS^®^ University Edition statistical software [27] (SAS Inst., Inc., Cary, NC, USA). The model included the main effects of hybrid of corn, drying temperature, inclusion of enzyme, and their interactions. The significant interactions were unfolded by the PROC GLM procedure of SAS using F or Tukey’s test. ANOSIM (Analysis of Similarity) test within the PRIMER 7 software package (PRIMER-E Ltd., Luton, UK) was used to analyze significant differences in microbial diversity communities between groups. All datasets were tested for normality using Shapiro–Wilk’s test (JMP Pro 11, SAS software Inc.) and then the Mann–Whitney test was performed (Prism v.8.2.1, GraphPad Software Inc., La Jolla, CA, USA), followed by a Dunn’s multiple comparison post-test. A level of significance of *p* ≤ 0.05 was adopted in all analyses.

## 3. Results

### 3.1. Growth Performance

The growth performance results are shown in Table 2. There was no interaction (*p* ≥ 0.05) between hybrids of corn, drying temperature, and inclusion of enzymes for any of the tested parameters. There was, however, an interaction between hybrids of corn and drying temperature (*p* = 0.0001), wherein the birds fed diets formulated with hybrid 2 dried at 110 °C had better FCR (1.342) from 1 to 21 d when compared to the birds fed the same hybrid dried at 80 °C (1.380). When corn was dried at 110 °C, a better FCR was observed in birds fed diets formulated with hybrid 2 when compared to those fed hybrid 3 (1.342 vs. 1.377; Table S1). Additionally, there was an interaction between enzyme and drying temperature, wherein the birds fed diets formulated with corn dried at 80 °C and supplemented with enzyme had better FCR from 1 to 7 d (*p* = 0.024; 1.254 vs. 1.356), and from 1 to 42 d (*p* = 0.04; 1.612 vs. 1.633; Table S2), compared to the non-supplemented birds.

Considering the main effects, it was observed that birds fed diets formulated with corn hybrid 3 had higher FI (*p* = 0.036) compared to those fed with corn hybrid 2. The use of corn dried at 110 °C led to lower FI (*p* = 0.002) and better FCR (*p* = 0.05) from 1 to 21 d, regardless of the corn hybrid or inclusion of enzyme. Additionally, the supplementation of the enzymatic blend improved BWG and FCR (*p* ≤ 0.001) from one to seven and one to 21 days of age (Table 2).

### 3.2. Intestinal Morphology

There was no interaction (*p* ≥ 0.05) between hybrids of corn, drying temperature, and inclusion of enzymes on the jejunum morphology parameters (Table 3). However, there was an interaction (*p* = 0.05) of the corn hybrid by enzyme inclusion, wherein birds fed diets formulated with corn hybrid 1 supplemented with enzymes showed higher VH at d 21 compared to non-supplemented birds (753.7 vs. 642.2 µm). Within the groups with or without enzyme supplementation, the birds receiving diets formulated with corn hybrids 1 and 3, respectively, had higher VH at d 21 (Table S3). There was an interaction between the hybrid of corn and drying temperature in which the birds fed diets formulated with corn hybrid 3 dried at 110 °C showed higher (*p* = 0.006) V:C at d 21 compared to the other hybrids dried at the same temperature, and with the same hybrid dried at 80 °C (Table S4). At d 42, birds fed diets formulated with corn hybrids 1 and 2 dried at 80 °C had higher V:C (*p* = 0.03) and AA (*p* = 0.02) compared to those fed the corn hybrid 3. Additionally, there was an interaction (*p* = 0.03) between enzyme and temperature wherein birds fed diets formulated with enzyme inclusion and corn dried at 80 °C had higher CD, and when dried at 110 °C had lower V:C (Table S5).

Considering the main effects, birds fed diets formulated with corn dried at 110 °C showed higher CD (*p* = 0.02) and V:C (*p* = 0.04) on d 21. However, on d 42, birds fed corn dried at 80 °C showed higher AA (*p* = 0.05). The inclusion of enzyme increased VH (*p* = 0.001), reduced CD (*p* = 0.001), and increased V:C and AA (*p* = 0.001) on d 21. On d 42, birds fed diets without enzyme supplementation showed higher AA (*p* = 0.02; Table 3).

### 3.3. Cecal Microbiota

**Figure 1:**
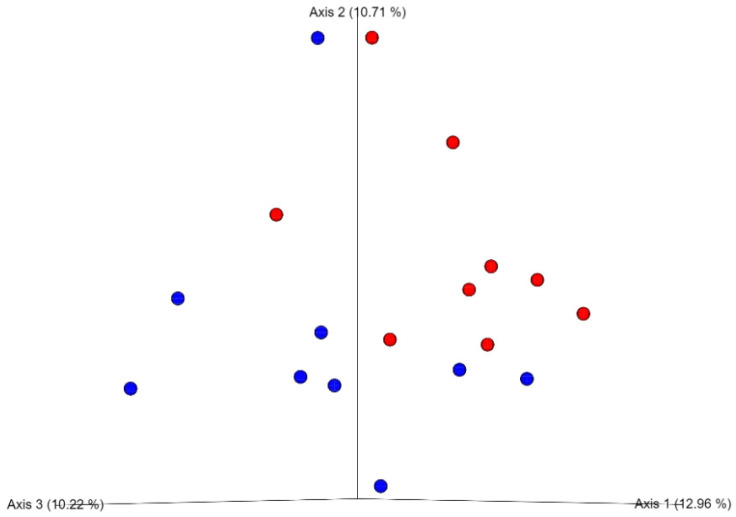
Principle coordinated analysis (PCoA) of the cecal microbiota of broilers at 21 days of age fed with or without inclusion of an enzymatic blend. Each point represents one sample (nine samples/treatment and a pool of cecal content from six birds/sample). Blue represents the samples from birds fed diets without enzyme supplementation, and red with enzyme supplementation.

The cecal microbiota analysis was focused on the effects of dietary inclusion of enzymes, as explained in the Material and Methods section. Overall, for the alpha diversity indices (variability within samples), there was no influence of the supplementation of enzyme on the Chao1 (*p* = 0.112) and Shannon (*p* = 0.401) indices wherein the average of the groups with or without supplementation was 145 vs. 133, and 6.17 vs. 6.07, respectively. However, for the beta diversity (variability between samples), there was visible clustering between birds supplemented and not supplemented with enzymes (Figure 1). While the clustering was visible, it did not reach statistical significance (ANOSIM, R = 0.126, *p* = 0.069).

The sequences were identified to the narrowest taxonomic rank possible. Overall, the microbiota composition was dominated by the phylum Firmicutes, Bacteroidetes, and Tenericutes, comprising 99% of the cecal microbiota. The phylum Firmicutes averaged at 95.66%, followed by Bacteroidetes (1.80%) and Tenericutes (1.52%). The phyla Proteobacteria, Actinobacteria, and Verrucomicrobia showed low frequency, below 1%. The only difference between the studied groups was observed at the phylum level for Tenericutes (*p* = 0.01) wherein enzyme supplementation increased its frequency (Table S6).

**Figure 2:**
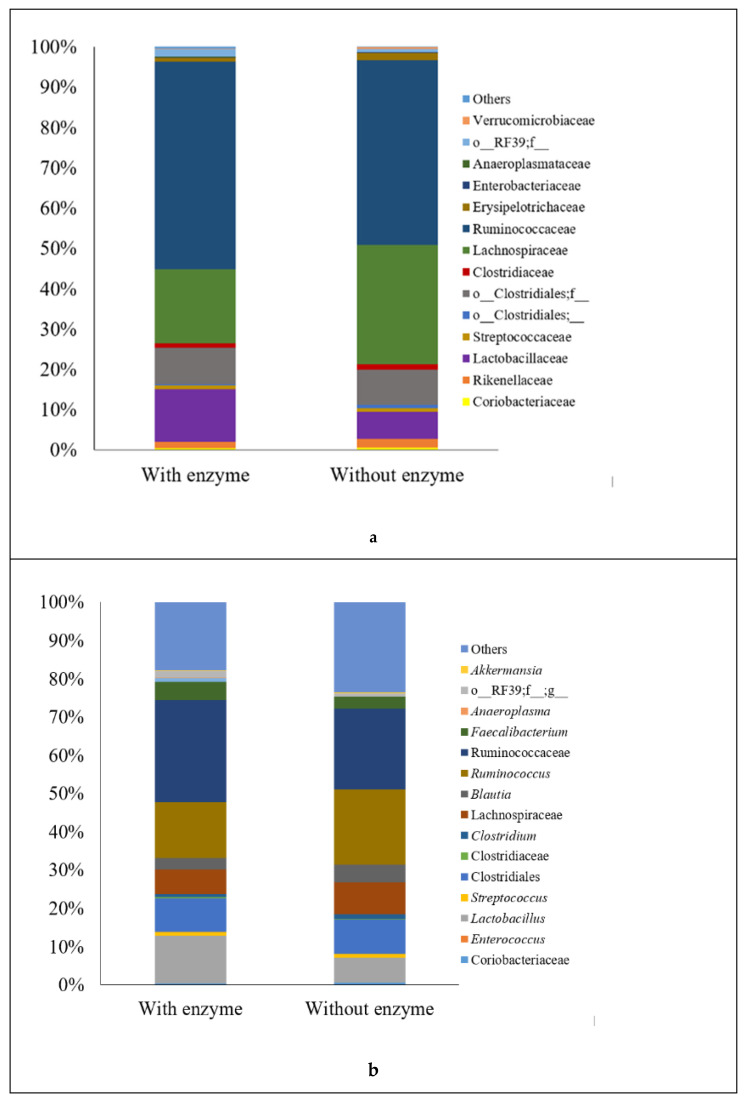
(**a**) Predominance of bacterial families and (**b**) bacterial genera present in the cecal microbiota of broilers at 21 days of age fed diets with or without inclusion of an enzymatic blend. The non-italicized groups represent bacteria that were identified to order- or family-level.

The microbiota of birds fed diets supplemented with enzymes showed an increase of bacteria belonging to the class Bacilli (*p* = 0.03; 14.33 vs. 7.17%) and Mollicutes (*p* = 0.01; 2.06 vs. 0.98%), and a trend toward a reduction of the class Clostridia (*p* = 0.09; 80.66 vs. 88.22%) (Table S6). Additionally, enzyme supplementation increased the abundance of the orders Lactobacillales (*p* = 0.04; 14.26 vs. 7.08%), Anaeroplasmatales (*p* = 0.03; 0.19 vs. 0%), and RF39 (*p* = 0.01; 1.82 vs. 0.8%) as well as their respective families, genus, and species. The family Lactobacillaceae, genus *Lactobacillus*, and species g_*Lactobacillus*; s_ increased in approximately 50% in birds supplemented with enzyme vs. non-supplemented birds (Figure 2; species data were not shown). The genus *Anaeroplasma* and specie g_*Anaeroplasma*; s_ were increased (*p* = 0.025; 0.19 vs. 0%) as well as the family o_RF39; F_, genus o_RF39; F_;G_, and species o_RF39; f_;g_,s_ (*p* = 0.014) by the enzyme supplementation. Additionally, the genus [*Ruminococcus*] showed an expressive reduction in birds fed diets containing the enzymatic complex (5.24 vs. 12.85%, with and without the enzyme, respectively; Figure 2; species data were not shown).

**Figure 3:**
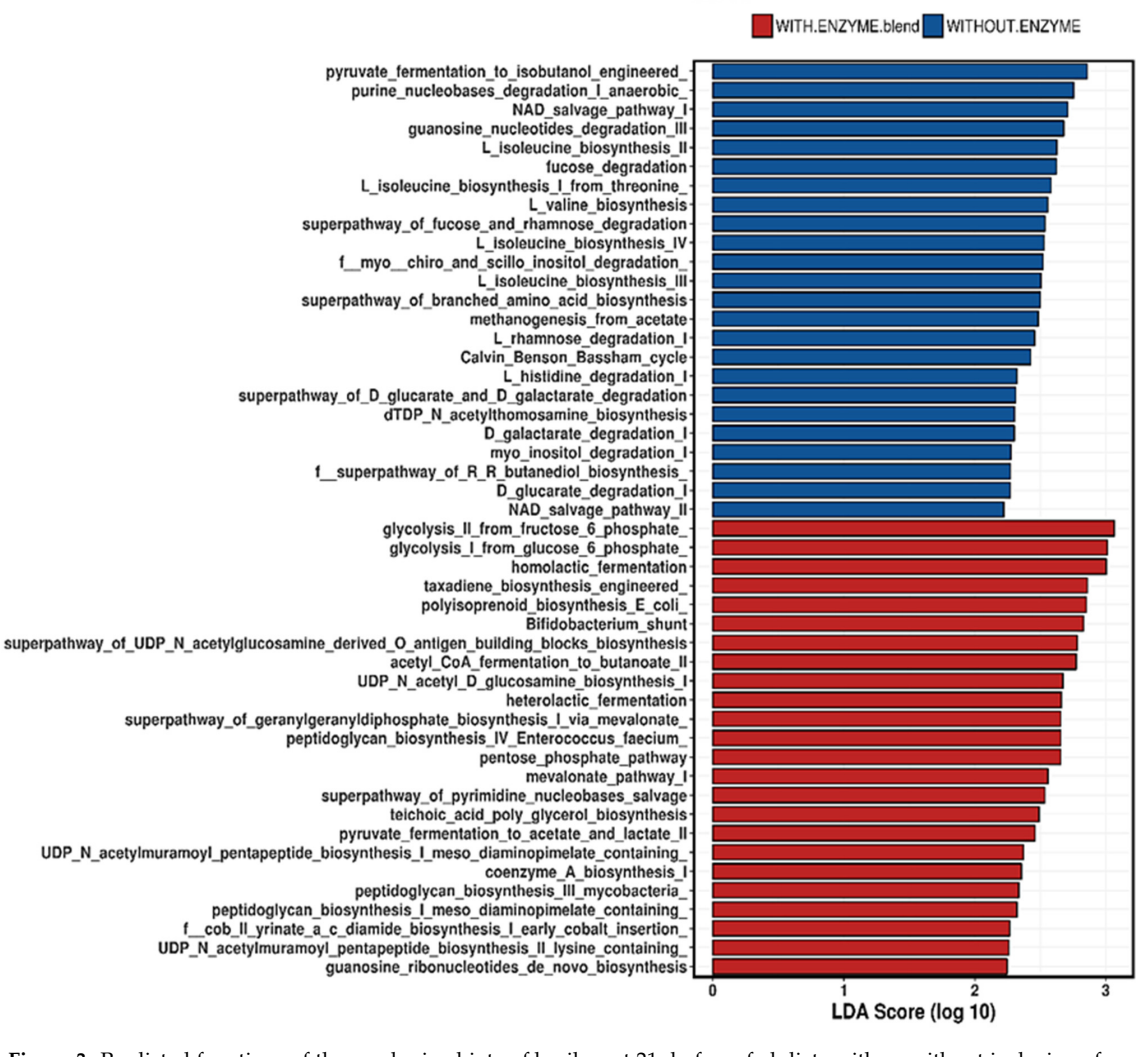
Predicted functions of the cecal microbiota of broilers at 21 d of age fed diets with or without inclusion of an enzymatic blend, identified by linear discriminant analysis (LDA) coupled with effect size (LEfSe) using default parameters.

The linear discriminant analysis (LDA) effect size (LEfSe) was performed in order to evaluate the differences on the predicted metabolic pathways found in the cecal microbiota (Figure 3). Overall, it was observed that 48 pathways were predicted to be different in the cecal microbiota of birds supplemented or not with enzymes. Of these, 24 were upregulated in the unsupplemented group and 24 were upregulated in the supplemented group. An enrichment of protein related pathways in the cecal microbiota of unsupplemented birds was observed, while a higher prevalence of carbohydrate-related pathways in the microbiota of birds supplemented with enzymes was observed.

### 3.4. Ileal Digestibility

There was an interaction between hybrids of corn, drying temperature, and inclusion of enzymatic blend for digestible energy (DE) (*p* < 0.001), coefficient of digestibility of dry matter (CDDM) (*p* < 0.001), crude protein (CDCP) (*p* = 0.042), and mineral matter (CDMM; Table 4) (*p* < 0.001). It was observed that birds fed diets supplemented with enzymes increased the DE in 11.98 and 7.47% for hybrid 2 and in 5.54 and 23.52% for hybrid 3 for the temperatures 80 °C and 110 °C, respectively. Additionally, regardless of the drying temperature, the dietary inclusion of enzymes increased the CDDM, CDCP, and CDMM in 10.05, 9.82, and 25.81% for hybrid 2 and in 14.70, 15.72, and 29.48% for hybrid 3, respectively (Table S7). For hybrid 1, an increase of 4.23, 6.21, and 46.89% was observed for CDDM, CDCP, and CDMM, respectively, when enzymes were included in the diets formulated with corn dried at 110 °C.

Moreover, it was observed that in diets without enzyme supplementation and formulated with corn hybrid 1, there was an increase of CDCP and CDMM when the corn was dried at 80 °C (71.67 vs. 68.08% and 38.10 vs. 22.65%, respectively). Similarly, for hybrid 3, the higher values of DE (2915 vs. 2465 kcal/kg) and CDDM (64.85 vs. 54.34%) were observed with a drying temperature of 80 °C. Within the diets containing enzymes, the effect of temperature was different between hybrids. For hybrid 1, higher DE (3084 vs. 2838 kcal/kg) and higher CDDM (67.58 vs. 62.50%) were observed with the drying temperature of 110 °C. In contrast, for hybrid 2, higher DE (3255 vs. 3025 kcal/kg) and higher CDDM (71.97 vs. 66.18%), CDCP (76.42 vs. 72.54%) and CDMM (52.27 vs. 38.60%), were observed when the corn was dried at 80 °C (Table S8).

### 3.5. Carcass Yield

Regarding the carcass and cuts yield data, there was no interaction between any of the factors evaluated (*p* > 0.05). Considering the main effects, birds supplemented with the enzymatic blend showed higher whole leg yield (*p* = 0.02; 27.42 vs. 26.97%) and lower percentage of abdominal fat (*p* = 0.03; 4.20 vs. 4.36%) at 42 days of age (Table S9).

## 4. Discussion

There are several corn hybrids available on the market with different physical-chemical characteristics. In the present study, we observed isolated effects of the corn hybrids in the presence of the enzymatic blend as well as the temperature used to dry the corn, which may be attributed to the genetic variation as the three hybrids used herein were grown at similar environmental conditions. The physical characteristics of corn as well as the amount of vitreous endosperm influence the extension of endosperm damage during the drying process, which seems to affect the hard endosperm more significantly [28]. The three hybrids evaluated had endosperm contents varying between 50 and 80% for both vitreous and floury endosperm (i.e., they were similar for this characteristic). The effects of the genetic variation of corn hybrids on the growth performance of broiler chickens are still unclear. Moore et al. [29] observed a positive correlation between the chemical characteristics of corn and the performance of broilers, without the influence of the physical characteristics. On the other hand, Kato et al. [30] did not observe any differences among the six different hybrids of corn on the energetic values for broiler chickens.

According to Jimenez-Moreno et al. [31], the differences found among studies and the drying temperatures impede the comparison of the results. The drying process is complex, and its effects depend on several parameters including initial grain moisture, dryer model, temperature, and drying time. Corn with a high initial moisture content is more susceptible to protein damage as a result of the thermic treatments [32]. Iji et al. [33] and Bhuiyan et al. [6] did not find an effect of the drying temperatures of 100 and 105 °C, respectively, on the ileal digestibility of crude energy. On the other hand, Kaczmarek et al. [28] observed a reduction of 100 kcal/kg of AME due to the lower ileal digestibility of starch and protein for a high drying temperature (140 °C). Therefore, the drying temperature of 110 °C used in the present study may have not been high enough to cause changes in the physical-chemical structure of the grains to consistently influence nutrient use and impair bird performance.

Although diets based on corn and soybean meal are not considered to promote high digesta viscosity, both ingredients contain NSP in their structure. The total content of NSP in corn varies from 6 to 9.7% [8,34], whereas soybean meal contains around 25% of NSP [35]. The hybrids of corn tested in the present work had, on average, an NSP content of 6.65%. Non-starch polysaccharides have a low digestibility due to an increased viscosity of the digesta, acting as a physical barrier and blocking the action of digestive enzymes, thus reducing the use of nutrients in the ingredients [36].

The use of exogenous enzymes such as xylanase may increase the access of endogenous enzymes to the proteins and starch present in the endosperm [37]. Romero et al. [38] showed a synergistic effect among amylase, xylanase, protease, and phytase in enhancing the AME_n_ content in the starter phase of chickens. In the present work, the dietary supplementation of enzymes could improve nutrient digestibility and growth performance of the chickens. In diets formulated with hybrids 2 and 3 and dried at 80 or 110 °C, the inclusion of enzymes increased the digestible energy and the digestibility coefficients of the nutrients when compared to the non-supplemented diets. The addition of the dietary blend improved BWG by 5% and FCR by 8% on d 7 and BWG by 3.8% and FCR by 3.2% on d 21. Regarding the overall experimental period (1–42 d), the addition of enzymes improved FCR by 1.3% when the diet was formulated with corn dried at 80 °C.

The consistent beneficial effect of enzymes on the performance of the birds from 1 to 21 d of age may be related to the fact that the gastrointestinal tract (GIT) of young chickens, mainly up to 10 d of age, is not fully matured, with lower activities of endogenous enzymes such as protease and lower nutrient digestibility [39]. According to Lee et al. [40], in young birds, digestion and absorption are still not adapted to the development of the GIT. Therefore, enhancing the accessibility of dietary nutrients in young chickens through the use of exogenous enzymes may lead to a superior use of nutrients and beneficial effects on growth performance [41].

The addition of exogenous enzymes such as amylase, xylanase, protease, and phytase to the diets may increase the nutrient digestibility due to the better access of the endogenous enzymes to the cellular content and hydrolysis of arabinoxylans of the cellular wall of the ingredients [37]. Xylanases are commonly used in the diets of broilers to diminish the antinutritional effect of NSP by reducing the viscosity of the digesta and the depolymerization of arabinoxylans of lower molecular weight. The excess of fiber in the GIT may damage the mucosa and reduce nutrient absorption, and dietary xylanase may mitigate this effect [42]. Besides the extra access of amylase to starch fractions due the action of xylanase, Córdova-Noboa et al. [43] demonstrated that the dietary supplementation of amylase can also increase energy use, nutrient digestibility, and broiler performance by reducing the impact on starch digestibility. According to a previous study, dietary supplementation of amylase seems to have an influence on nutrient digestibility, reducing the variability in the energy value of diets formulated with poorly digestible corn [44]. Zhang et al. [45] showed that the use of exogenous enzymes increased the ileal and total digestibility of crude protein and starch and improved the growth performance of broilers. The use of enzymatic blends has been shown to be efficient in diets based on corn and soybean meal for broilers, improving performance and nutrient digestibility [41,46].

The better results obtained in the present study from d 1 to 21, with the addition of exogenous enzymes, may be associated with improved absorptive capacity of the intestinal mucosa. According to Munyaka et al. [47], changes in the intestinal microbiota may alter the structure of the mucosa and influence nutrient absorption, which can partially explain the differences in BWG observed in the present study. Considering that the effects of the inclusion of the enzymatic blend on the growth performance and intestinal morphology were consistent from 1 to 21 d, we opted to evaluate the cecal microbiota at d 21 in birds receiving enzyme supplementation or not, regardless of the hybrid or drying temperature. Although not significant, the results of the microbiota diversity (higher Chao1 and Shannon Indices) in birds supplemented with enzymes agreed with the changes in the abundance of the bacterial groups.

The diversity and the different functions exerted by the microbiota are related to the health and productivity of the animals [48,49], and the nature and extension of the microbial changes may determine if there will be effects on the performance of the birds [50]. For instance, antibiotics, probiotics, organic acids, and enzymes as well as diet processing, management, and stress may affect the composition of the microbiota [51,52,53]. On the phylum level, the ratio between Firmicutes and Bacteroidetes may be indicative of a better feed efficiency. The phylum Firmicutes is related to better performance and use of dietary nutrients, whereas the phylum Bacteroidetes is related to lower digestive capacity and absorption of nutrients by the host [54,55].

The variation in the abundance of bacterial phyla may be associated with the type of diet [56]. Diets with high concentrations of fiber and low in fat increase Bacteroidetes, while diets with low fiber and high fat may enhance the percentage of Firmicutes [40], which is in agreement with the findings observed in the present study as the experimental diets were based on corn and soybean meal with low fiber concentrations. In a previous study, enzyme supplementation of broiler diets led to a microbiota dominated by Firmicutes and Tenericutes [57]. In the present study, the phylum Tenericutes increased by 100% in birds supplemented with enzymes, along with the class Mollicutes and the order RF39 present in this phylum. According to [58], the class Mollicutes is parasitic and does not have a cellular wall. However, this class was almost formed by the order RF39, which is considered not pathogenic [57]. Additionally, belonging to the phylum Tenericutes, the group Anaplasmatales increased by 19% as well as its family, genus, and species in birds supplemented with enzymes, while the birds not supplemented with enzymes did not present these bacteria. Beller et al. [59] reported that bacteria in the genus *Anaeroplasma* have anti-inflammatory properties, with the capacity to upregulate TGF-β, a regulatory cytokine, improving the intestinal barrier and increasing mucosal IgA. Therefore, bacteria in the genus *Anaeroplasma* may be considered probiotic, being able to prevent and treat chronic inflammation [59].

The cecal microbiota contains most of the resident bacteria of the GIT of domestic birds [60,61], dominated mainly by bacteria of the class Clostridia [49], which agrees with the finding of the present study (84.44% of Clostridia). The main genera of this class include *Ruminococcus*, *Faecalibacterium*, and *Eubacterium* [62,63]. In the present study, enzymatic supplementation led to a 50% reduction of the genus *Ruminococcus*, which degrades structural carbohydrates that are not hydrolyzed by the endogenous enzymes of the host [47] (i.e., cellulose and resistant starch). The reduction in the frequency of this genus may be related to the action of the exogenous enzymes added to the diet, which solubilize the non-digested carbohydrates and, consequently, reduce the substrates available for these bacteria.

According to Jozefiak et al. [60], differences in the dietary composition, use of enzymes (isolated or in association), and their mode of action, varying with the available substrates, may favor changes of the composition of the digesta entering the ceca. The ceca are the main site of bacterial fermentation in the intestine of broilers and the only compartment where it would be possible to use part of the structural carbohydrates not degraded by the endogenous enzymes [64]. Therefore, the changes in the luminal composition promoted by the dietary enzymes may alter the substrate at the cecal level and its microbiota as well the microbiota composition along the entire GIT. The modulation of the cecal microbiota observed in the present study may have changed the digestive process and nutrient use by the animal. Additionally, the change in digesta composition reaching the ceca and, consequently, its microbiota composition, may have favored the modulation of the predicted functions performed by the microbiota. The microbiota of birds not supplemented with enzymes was enriched with metabolic pathways related to protein metabolism, possibly increasing the loss of endogenous and dietary nitrogen in the excreta [65]. On the other hand, dietary enzymes increased the frequency of pathways linked to the metabolism of carbohydrates, which may also explain the better results in terms of growth performance.

Regarding the carcass yield parameters, there was a reduction in the percentage of abdominal fat and an increase in the whole leg yield when the diets were supplemented with exogenous enzymes. These results are possibly due to the balance promoted by the enzymes in the availability and use of nutrients present in the ingredients [66]. It has been demonstrated that carbohydrates have a protein-sparing effect [67] and that amylase may increase starch digestibility [68], thereby affecting carcass characteristics and increasing nitrogen retention. Additionally, the starch source may influence the expression of genes of enzymes that regulate glycogen synthesis (glycogen synthase kinase-3 β) and fat synthesis (fatty acid synthase) [69]. This effect can lead to the hypothesis that dietary enzymes influence the availability of nutrients and the expression of enzymes related to the carbohydrate and lipid metabolisms, which can affect carcass composition and yield.

## 5. Conclusions

In conclusion, the drying temperature and the different hybrids of corn evaluated herein may not have caused changes in the physical-chemical structure of the grains to consistently influence the use of nutrients and impair the performance of the birds. The enzymatic supplementation of amylase, xylanase, and protease led to better growth performance, which may be due to the improved intestinal morphology, better ileal digestibility, and modification of the diversity, composition, and predicted function of the cecal microbiota at 21 days of age of broiler chickens.

## Figures and Tables

**Table 1:** Composition of the experimental diets (1–7, 8–21, and 22–42 days (d) of age).

Ingredient, g/kg	1–7 d	8–21 d	22–42 d
Corn	585.0	623.0	668.0
Soybean meal, 46%	348.0	317.0	268.0
Soybean oil	15.2	15.8	25.8
Limestone	11.4	10.9	9.54
Monocalcium phosphate	17.9	14.1	10.99
Salt (NaCl)	5.07	4.81	4.49
Lysine sulfate, 50.7%	5.43	4.26	4.04
DL- Methionine, 99%	4.03	3.26	2.81
L-Threonine, 99%	1.80	1.22	0.97
L-Valine, 93.5%	1.42	0.81	0.71
Choline chloride, 60%	0.60	0.60	0.60
Premix vitaminic ^1^	1.50	1.50	1.50
Premix mineral ^2^	0.50	0.50	0.50
Antioxidant ^3^	0.20	0.20	0.20
Growth promoter ^4^	0.05	0.05	0.05
Anticoccidian ^5^	0.60	0.60	0.60
Inert ^6^	0.40	0.40	0.40
Total	100.00	100.00	100.00
Calculated nutritional content (as dry matter)			
Metabolizable Energy, Kcal/kg	2950	3000	3125
Crude protein, %	22.20	20.80	18.75
Calcium, %	0.920	0.819	0.685
Available phosphorus, %	0.470	0.391	0.320
Digestible lysine, %	1.310	1.174	1.044
Digestible Met + Cys, %	0.944	0.843	0.762
Digestible threonine, %	0.852	0.763	0.678
Digestible tryptophan, %	0.223	0.214	0.188
Chloride, %	0.354	0.340	0.321
Sodium, %	0.220	0.210	0.197
Potassium, %	0.903	0.849	0.760

^1^ Vitamins provided per kg of feed: vitamin A, 1350 UI; vitamin D3, 3750 UI; vitamin E, 30 UI; vitamin K3, 3.75 mg; vitamin B1, 2.25 mg; vitamin B2, 9 mg; vitamin B6, 4.5 mg; vitamin B12, 18 mg; pantothenic acid, 18 mg; niacin, 37.5 mg; folic acid, 1.2 mg; biotin, 90 mcg; selenium, 375 mcg; ^2^ Trace minerals provided per kg of the feed: copper, 10 mg; iron, 50 mg; manganese, 240 mg; cobalt, 1 mg; iodine, 1 mg; zinc, 50 mg; ^3^ BHT = butylated hydroxytoluene; ^4^ Avilamycin; ^5^ Salinomycin sodium; ^6^ The enzymatic blend (per kg of diet) contained 80 KNU (kilo-Novo α-amilase units) (RONOZYME^®^ HiStarch), 100 FXU (fungal β-xylanase units) (RONOZYME^®^ WX), and 15,000 PROT (protease units) (RONOZYME^®^ ProAct), and all the diets were formulated with the inclusion of phytase (1000 FYT (phytase units per kg of feed), RONOZYME^®^ Hiphos).

**Table 2:** Performance of broilers fed diets containing different corn hybrids, dried at two temperatures, and with or without the inclusion of an enzymatic blend.

Treatments	1–7 d			1–21 d			1–42 d		
	BWG, g	FI, g	FCR	BWG, g	FI, g	FCR	BWG, g	FI, g	FCR
Hybrids									
1	117.5	153.2	1.306	797.3	1087.4	1.364	2832.9	4575.4 ^ab^	1.615
2	117.7	152.2	1.295	793.4	1079.7	1.362	2806.4	4552.4 ^b^	1.623
3	117.2	151.8	1.297	801.4	1095.5	1.367	2862.6	4640.4 ^a^	1.622
Temperature									
80 °C	117.6	153.0	1.305	800.9	1096.2 ^a^	1.370 ^b^	2838.5	4603.9	1.622
110 °C	117.3	151.7	1.294	793.9	1078.7 ^b^	1.359 ^a^	2829.9	4575.3	1.617
Enzyme									
With enzyme	119.4^a^	151.2	1.267 ^b^	812.3 ^a^	1091.1	1.343 ^b^	2843.6	4591.6	1.615
Without enzyme	115.4^b^	153.5	1.332 ^a^	782.3 ^b^	1083.	1.386 ^a^	2824.7	4587.9	1.625
SEM	0.460	0.842	0.009	2.653	2.968	0.004	9.749	14.541	0.003
Source of variation	*p*-Values								
Hybrid, Hyb	0.888	0.786	0.835	0.323	0.081	0.713	0.070	0.036	0.568
Temperature, Temp	0.684	0.422	0.529	0.107	0.002	0.045	0.637	0.298	0.380
Enzyme, ENZ	<0.001	0.183	0.0002	<0.001	0.181	<0.001	0.323	0.848	0.115
Hyb*ENZ	0.588	0.709	0.986	0.776	0.935	0.382	0.925	0.772	0.825
Hyb*Temp	0.307	0.086	0.073	0.292	0.093	<0.001	0.678	0.138	0.223
ENZ*Temp	0.303	0.229	0.024	0.123	0.702	0.072	0.547	0.656	0.040
Hyb*ENZ*Temp	0.342	0.878	0.399	0.748	0.909	0.843	0.816	0.871	0.625

BWG = Body weight gain; FI = Feed intake; FCR = Feed conversion ratio; SEM = standard error of the mean; Means followed by distinct letters in the same column are different (*p* ≤ 0.05) by Tukey’s or F test; The enzymatic blend contained 80 KNU/kg of amylase (RONOZYME^®^ HiStarch), 100 FXU/kg of xylanase (RONOZYME^®^ WX), and 15,000 PROT/kg of protease (RONOZYME^®^ ProAct), and all diets were formulated with the inclusion of phytase (1000 FYT/kg of feed, RONOZYME^®^ Hiphos).

**Table 3:** Villus height (μm), crypt depth (μm), villus:crypt ratio, and area of absorption (µm^2^) of the jejunum of broilers fed diets containing different corn hybrids, dried at two temperatures, and with or without the inclusion of an enzymatic blend.

Treatments	21 d				42 d			
	Villus Height	Crypt Depth	Villus:Crypt Ratio	Area of Absorption	Villus Height	Crypt Depth	Villus:Crypt Ratio	Area of Absorption
Hybrids								
1	712.37	66.98	10.88	13.89	744.48	68.64	11.31	13.06
2	704.75	65.63	11.03	14.48	788.23	72.32	11.49	13.20
3	725.00	65.53	11.43	14.91	721.76	70.34	10.82	12.80
Temperature								
80 °C	712.01	66.97 ^a^	10.90 ^b^	14.12	764.24	70.96	11.27	13.34 ^a^
110 °C	716.16	65.06 ^b^	11.35 ^a^	14.79	736.17	70.06	11.10	12.63 ^b^
Enzyme								
With enzyme	738.79 ^a^	63.05 ^b^	12.11 ^a^	15.32 ^a^	734.03	71.28	10.90	12.40 ^b^
Without enzyme	686.83 ^b^	69.37 ^a^	10.07 ^b^	13.57 ^b^	768.59	69.77	11.49	13.64 ^a^
SEM	9.107	0.830	0.183	0.216	15.901	1.169	0.271	0.240
Source of variation	*p*-Values							
Hybrid, Hyb	0.652	0.735	0.451	0.165	0.243	0.661	0.417	0.901
Temperature, Temp	0.685	0.017	0.040	0.072	0.250	0.460	0.818	0.048
Enzyme, ENZ	0.001	0.001	<0.001	0.001	0.354	0.346	0.584	0.015
Hyb*ENZ	0.048	0.254	0.852	0.066	0.767	0.448	0.118	0.920
Hyb*Temp	0.155	0.172	0.006	0.308	0.340	0.092	0.031	0.023
ENZ*Temp	0.173	0.733	0.201	0.544	0.748	0.025	0.008	0.334
Hyb*ENZ*Temp	0.091	0.352	0.724	0.633	0.718	0.287	0.398	0.135

^a,b^ Means followed by distinct letters in the same column are different (*p* ≤ 0.05) by the F test; SEM = standard error of the mean; The enzymatic blend contained 80 KNU/kg of amylase (RONOZYME^®^ HiStarch), 100 FXU/kg of xylanase (RONOZYME^®^ WX), and 15,000 PROT/kg of protease (RONOZYME^®^ ProAct), and all diets were formulated with the inclusion of phytase (1000 FYT/kg of feed, RONOZYME^®^ Hiphos).

**Table 4:** Average values of digestible energy (kcal/kg) and digestibility coefficients (%) of dry matter, crude protein and mineral matter determined in broilers fed diets containing different corn hybrids, dried at two temperatures, and with or without the inclusion of an enzymatic blend.

Treatments	Digestible Energy	CDDM	CDCP	CDMM
Hybrids				
1	2970	65.02	70.18 ^A^	34.9
2	2990	65.68	70.91 ^A^	39.8
3	2918	64.63	65.98 ^B^	37.2
Temperature				
80 °C	2992 ^a^	66.00 ^a^	69.71	37.6
110 °C	2930 ^b^	64.27 ^b^	68.45	37.0
Enzyme				
With enzyme	3085 ^a^	67.99 ^a^	72.20 ^a^	42.8 ^a^
Without enzyme	2832 ^b^	62.18 ^b^	65.86 ^b^	31.7 ^b^
SEM	24.393	0.548	0.614	1.154
Source of variation	*p*-Values			
Hybrid, Hyb	0.195	0.552	<0.001	0.112
Temperature, Temp	0.045	0.012	0.206	0.827
Enzyme, ENZ	<0.001	<0.001	<0.001	<0.001
Hyb*ENZ	<0.001	<0.001	<0.001	0.678
Hyb*Temp	<0.001	<0.001	0.385	0.025
ENZ*Temp	<0.001	<0.001	0.335	0.971
Hyb*ENZ*Temp	<0.001	<0.001	0.042	<0.001

^A,B/a,b^ Means followed by distinct capital and small letters in the same column are different (*p* ≤ 0.05) by Tukey’s and F’s test, respectively; CDDM = coefficient of digestibility of dry matter; CDCP = coefficient of digestibility of crude protein; CDMM = coefficient of digestibility of mineral matter; SEM = standard error of the mean; The enzymatic blend contained 80 KNU/kg of amylase (RONOZYME^®^ HiStarch), 100 FXU/kg of xylanase (RONOZYME^®^ WX), and 15,000 PROT/kg of protease (RONOZYME^®^ ProAct), and all diets were formulated with the inclusion of phytase (1000 FYT/kg of feed, RONOZYME^®^ Hiphos).

## Data Availability

The data presented in this study are available on reasonable request from the corresponding author.

## Supplementary Materials

The following are available online at https://www.mdpi.com/2076-2615/11/3/643/s1: Table S1: Feed conversion ratio of broilers from 1 to 21 days fed diets containing different corn hybrids dried at two temperatures, Table S2: Feed conversion ratio of broilers fed diets containing corn dried at two temperatures and with or without inclusion of the enzymatic blend, Table S3: Villus height of jejunum of broilers at 21 days fed diets containing different corn hybrids and with or without inclusion of the enzymatic blend, Table S4: Villus:crypt ratio and area of absorption of jejunum of broilers fed diets containing different corn hybrids dried at two temperatures, Table S5: Crypt depth and villus:crypt ratio of broilers at 42 days fed diets containing corn dried at two temperatures and with or without inclusion of enzymatic blend, Table S6: Abundance of phyla, classes, and bacterial orders found in the cecal microbiota of broilers at 21 days of age fed diets with or without inclusion of the enzymatic blend, Table S7: Digestible energy and the digestibility coefficients of dry matter, crude protein, and mineral matter determined with broilers fed diets containing different corn hybrids, dried at different temperatures, and with or without the inclusion of the enzymatic blend, Table S8: Digestible energy and the digestibility coefficients of dry matter, crude protein, and mineral matter determined with broilers fed diets containing different corn hybrids, with or without the inclusion of the enzymatic blend, and dried at different temperatures, Table S9: Carcass yield, cuts yield, and percentage of abdominal fat of broilers at 42 days of age fed diets containing different corn hybrids, dried at two temperatures, and with or without inclusion of the enzymatic blend.
