# Supplementary material for: Influence of Enzyme Supplementation in the Diets of Broiler Chickens Formulated with Different Corn Hybrids Dried at Various Temperatures
Source: Animals (Basel). 2021 Feb 28;11(3):643. doi: 10.3390/ani11030643 (PMC7997286; doi:10.3390/ani11030643)
Supplement: Supplementary file 1 [file animals-11-00643-s001.pdf]

animals

MDPI

Supplementary Materials: Influence of Enzyme Supplementation in the Diets of Broiler Chickens Formulated with Different Corn Hybrids Dried at Various Temperatures

Franciele C. N. Giacobbo, Cinthia Eyng, Ricardo V. Nunes, Cleison de Souza, Levy V. Teixeira, Rachel Pilla, Jan S. Suchodolski and Cristiano Bortoluzzi

Table S1. Feed conversion ratio of broilers, from 1 to 21 days, fed diets containing different corn hybrids dried at two temperatures.

Hybrid	Temperature	
	80 °C	110 °C
1	1.371	1.357 [AB]
2	1.380[a]	1.342 [Bb]
3	1.358	1.377[A]

[A,B/a,b]Means followed by distinct capital letters, in the same column, and small letters, in the same row, are different ($p \leq 0.05$) by Tukey's or F's test, respectively.

Table S2. Feed conversion ratio of broilers fed diets containing corn dried at two temperatures and with or without inclusion of enzymatic blend.

	1–7 Days		1–42 Days	
	Temperature		Temperature	
Enzyme	80 °C	110 °C	80 °C	110 °C
With enzyme	1.254[B]	1.281	1.612[B]	1.619
Without enzyme	1.356[A]	1.308	1.633[Aa]	1.616[b]

[A,B/a,b]Means followed by distinct capital letters, in the same column, and small letters, in the same row, are different ($p \leq 0.05$) by F test; The enzymatic blend contained 80 KNU/kg of amylase (RONOZYME® HiStarch), 100 FXU/kg of xylanase (RONOZYME® WX), and 15,000 PROT/kg of protease (RONOZYME® ProAct), and all the diets were formulated with the inclusion of phytase (1,000 FYT/kg of feed, RONOZYME® Hiphos).

Table S3. Villus height (μm) of jejunum of broilers, at 21 days, fed diets containing different corn hybrids and with or without inclusion of enzymatic blend.

Enzyme	Hybrid 1	Hybrid 2	Hybrid 3
With enzyme	753.68 [Aa]	725.05 [b]	737.17 [b]
Without enzyme	642.15 [Bb]	680.09 [ab]	716.89 [a]

[A,B/a,b]Means followed by distinct capital letters, in the same column, and small letters, in the same row, are different ($p \leq 0.05$) by F's or Tukey's test, respectively; The enzymatic blend contained 80 KNU/kg of amylase (RONOZYME® HiStarch), 100 FXU/kg of xylanase (RONOZYME® WX), and 15,000 PROT/kg of protease (RONOZYME® ProAct), and all the diets were formulated with the inclusion of phytase (1 000 FYT/kg of feed, RONOZYME® Hiphos).

Table S4. Villus:crypt ratio and area of absorption (μm^2) of jejunum of broilers fed diets containing different corn hybrids dried at two temperatures.

	21 Days		42 Days			
	Villus:crypt ratio		Villus:crypt ratio		Area of absorption	
Hybrids	80 °C	110 °C	80 °C	110 °C	80°C	110 °C
1	10.94	10.81[B]	12.27 [Aa]	10.26 [b]	13.92 [Aa]	12.12 [b]
2	10.97	11.08 [B]	11.58 [A]	11.40	14.00 [Aa]	12.40 [b]
3	10.82[b]	12.27 [Aa]	10.32 [B]	11.53	12.38 [B]	13.42

A,B/a,bMeans followed by distinct capital letters, in the same column, and small letters, in the same row, are different ($p \leq 0.05$) by Tukey's or F's test, respectively.

Table S5. Crypt depth (μm) and villus:crypt ratio of broilers, at 42 days, fed diets containing corn dried at two temperatures and with or without inclusion of enzymatic blend.

Enzyme	Crypt Depth		Villus:Crypt Ratio	
	**80 °C**	**110 °C**	**80 °C**	**110 °C**
With enzyme	74.83 Aa	67.74 b	10.29 B	11.51
Without enzyme	67.44 Bb	72.92 a	12.16 A	10.63

A,B/a,bMeans followed by distinct capital letters, in the same column, and small letters, in the same row, are different ($p \leq 0.05$) by F test; The enzymatic blend contained 80 KNU/kg of amylase (RONOZYME® HiStarch), 100 FXU/kg of xylanase (RONOZYME® WX), and 15,000 PROT/kg of protease (RONOZYME® ProAct), and all the diets were formulated with the inclusion of phytase (1,000 FYT/kg of feed, RONOZYME® Hiphos).

Table S6. Abundance of phyla, classes and bacterial orders found in the cecal microbiota of broilers, at 21 days of age, fed diets with or without inclusion of enzymatic blend.

	**With Enzyme**		**Without Enzyme**		
	Average	Interval	Average	Interval	*p*-Value
Phylum					
Actinobacteria	0.38	0.24–0.92	0.64	0.29–1.40	0.161
Bacteroidetes	1.58	0.87–2.48	2.01	0.00–4.64	0.286
Firmicutes	94.83	92.26–97.98	96.49	91.77–97.35	0.863
Proteobacteria	0.07	0–0,31	0.12	0–2.89	0.262
Tenericutes	2.06	0.23–3.95	0.98	0.13–2.20	0.014
Verrucomicrobia	0.16	0–3.68	0.35	0–1.41	0.813
Class					
Coriobacteriia	0.38	0.24–0.92	0.61	0.29–1.40	0.162
Bacteroidia	1.58	0.87–2.48	2.01	0–4.64	0.286
Bacilli	14.33	7.07–25.63	7.17	2.55–14.87	0.032
Clostridia	80.66	69.62–85.41	88.22	75.33–90.56	0.094
Erysipelotrichi	0.98	0.41–3.30	1.85	0.62–3.30	0.489
Gammaproteobacteria	0.07	0–0.31	0.12	0–2.87	0.262
Mollicutes	2.06	0.23–3.95	0.98	0.13–2.20	0.014
Verrucomicrobiae	0.16	0–3.68	0.35	0–1.41	0.813
Order					
Coriobacteriales	0.38	0.24–0.92	0.61	0.29–1.40	0.162
Bacteroidales	1.58	0.87–2.48	2.01	0–4.64	0.286
Bacillales	0.11	0.02–0.16	0.06	0-–0.18	0.230
Lactobacillales	14.26	6.99–25.52	7.08	2.49–14.87	0.040
Clostridiales	80.66	69.62–85.41	88.22	75.33–90.56	0.094
Erysipelotrichales	0.98	0.41-–3.30	1.85	0.62–3.30	0.489
Enterobacteriales	0.07	0–0.31	0.12	0–2.87	0.262
Anaeroplasmatales	0.19	0.03–0.64	0	0–0.84	0.025
RF39	1.82	0.20–3.76	0.80	0.13–2.20	0.014
Verrucomicrobiales	0.16	0–3.68	0.35	0–1.41	0.813

The enzymatic blend contained 80 KNU/kg of amylase (RONOZYME® HiStarch), 100 FXU/kg of xylanase (RONOZYME® WX), and 15,000 PROT/kg of protease (RONOZYME® ProAct), and all the diets were formulated with the inclusion of phytase (1,000 FYT/kg of feed, RONOZYME® Hiphos).

Table S7. Digestible energy (kcal/kg) and the digestibility coefficients (%) of dry matter, crude protein and mineral matter determined with broilers fed diets containing different corn hybrids, dried at different temperatures, and with or without the inclusion of blend enzymatic.

Hybrids	Digestible energy				CDDM			
	80 °C		110 °C		80 °C		110 °C	
	with	without	with	without	with	without	with	without
1	2838 ^{a}	2976 ^{a}	3084 ^{a}	2980 ^{a}	62.50 ^{a}	65.28 ^{a}	67.58 ^{a}	64.72 ^{b}
2	3255 ^{a}	2865 ^{b}	3025 ^{a}	2799 ^{b}	71.97 ^{a}	63.02 ^{b}	66.18 ^{a}	61.26 ^{b}
3	3086 ^{a}	2915 ^{b}	3223 ^{a}	2465 ^{b}	68.18 ^{a}	64.85 ^{a}	71.56 ^{a}	54.34 ^{b}

Hybrids	CDCP				CDMM			
	80 °C		110 °C		80 °C		110 °C	
	with	without	with	without	with	without	with	without
1	68.37 ^{a}	71.67 ^{a}	72.59 ^{a}	68.08 ^{b}	36.08 ^{a}	38.10 ^{a}	42.65 ^{a}	22.65 ^{b}
2	76.42 ^{a}	67.85 ^{b}	72.54 ^{a}	66.48 ^{b}	52.27 ^{a}	30.41^{b}	38.60 ^{a}	37.01 ^{a}
3	72.21^{a}	60.84 ^{b}	71.06 ^{a}	59.92 ^{b}	40.57 ^{a}	26.69 ^{b}	46.27 ^{a}	34.54 ^{b}

CDDM = coefficient of digestibility of dry matter; CDCP = coefficient of digestibility of crude protein; CDMM = coefficient of digestibility of mineral matter; ^{a,b}Means within each group (drying temperature - 80 and 110 ° C) for each corn hybrid, followed by different letters on the row are different ($p \leq 0.05$) by F test; The enzymatic blend contained 80 KNU/kg of amylase (RONOZYME® HiStarch), 100 FXU/kg of xylanase (RONOZYME® WX), and 15,000 PROT/kg of protease (RONOZYME® ProAct), and all the diets were formulated with the inclusion of phytase (1,000 FYT/kg of feed, RONOZYME® Hiphos).

Table S8. Digestible energy (kcal/kg) and the digestibility coefficients (%) of dry matter, crude protein and mineral matter determined with broilers fed diets containing different corn hybrids, with or without the inclusion of enzymatic blend, and dried at different temperatures.

Hybrids	Digestible Energy				CDDM			
	with		without		with		without	
	80 °C	110 °C	80 °C	110 °C	80 °C	110 °C	80 °C	110 °C
1	2838 ^{b}	3084 ^{a}	2976 ^{a}	2980 ^{a}	62.50 ^{b}	67.58 ^{a}	65.28 ^{a}	64.72 ^{a}
2	3255 ^{a}	3025 ^{b}	2865 ^{a}	2799 ^{a}	71.97 ^{a}	66.18 ^{b}	63.01^{a}	61.26 ^{a}
3	3086 ^{a}	3223 ^{a}	2915 ^{a}	2465 ^{b}	68.18 ^{a}	71.56 ^{a}	64.85 ^{a}	54.34 ^{b}

Hybrids	CDCP				CDMM			
	with		without		with		without	
	80 °C	110 °C	80 °C	110 °C	80 °C	110 °C	80 °C	110 °C
1	68.36 ^{a}	72.59 ^{a}	71.67 ^{a}	68.08 ^{b}	36.08 ^{a}	42.65 ^{a}	38.10 ^{a}	22.65 ^{b}
2	76.42 ^{a}	72.54 ^{b}	67.85 ^{a}	66.48 ^{a}	52.27 ^{a}	38.60 ^{b}	30.41^{a}	37.01^{a}
3	72.21^{a}	71.06 ^{a}	60.84 ^{a}	59.92 ^{a}	40.57 ^{a}	46.27 ^{a}	26.69 ^{a}	34.54 ^{a}

CDDM = coefficient of digestibility of dry matter; CDCP = coefficient of digestibility of crude protein; CDMM = coefficient of digestibility of mineral matter; ^{a,b}Means within each group (with or without inclusion of enzymatic blend) for each corn hybrid, followed by different letters in the row are different ($p \leq 0.05$) by F test; The enzymatic blend contained 80 KNU/kg of amylase (RONOZYME® HiStarch), 100 FXU/kg of xylanase (RONOZYME® WX), and 15,000 PROT/kg of protease (RONOZYME® ProAct), and all the diets were formulated with the inclusion of phytase (1,000 FYT/kg of feed, RONOZYME® Hiphos).

Table S9. Carcass yield, cuts yield (%) and percentage of abdominal fat (%) of broilers, at 42 days of age, fed diets containing different corn hybrids, dried at two temperatures and with or without inclusion of enzymatic blend.

Treatments	Carcass	Breast	Leg	Wings	Abdominal Fat
Hybrids					
1	77.07	37.14	27.10	9.27	4.38
2	77.42	37.35	27.19	9.22	4.26
3	77.43	37.17	27.31	9.18	4.20
Temperature					
80 °C	77.42	37.45	27.10	9.27	4.30
110 °C	77.19	36.98	27.30	9.17	4.25
Enzyme					
With enzyme	77.34	37.05	27.42 [a]	9.24	4.20 [b]
Without enzyme	77.26	37.38	26.97 [b]	9.21	4.36 [a]
SEM	1.729	4.169	3.714	3.507	8.868
Source of variation			*p*-Values		
Hybrid, Hyb	0.468	0.818	0.683	0.500	0.120
Temperature, Temp	0.380	0.108	0.298	0.112	0.516
Enzyme, ENZ	0.763	0.263	0.022	0.665	0.025
Hyb*ENZ	0.721	0.218	0.321	0.893	0.755
Hyb*Temp	0.794	0.067	0.838	0.070	0.892
ENZ*Temp	0.675	0.448	0.069	0.207	0.931
Hyb*ENZ*Temp	0.798	0.280	0.978	0.622	0.580

[a,b]Means followed by distinct letters in the same column are different ($p \leq 0.05$) by F test; The enzymatic blend contained 80 KNU/kg of amylase (RONOZYME® HiStarch), 100 FXU/kg of xylanase (RONOZYME® WX), and 15,000 PROT/kg of protease (RONOZYME® ProAct), and all the diets were formulated with the inclusion of phytase (1,000 FYT/kg of feed, RONOZYME® Hiphos).
